# Human Sentiment and Activity Recognition in Disaster Situations Using Social Media Images Based on Deep Learning

**DOI:** 10.3390/s20247115

**Published:** 2020-12-11

**Authors:** Amin Muhammad Sadiq, Huynsik Ahn, Young Bok Choi

**Affiliations:** 1 Department of Robot System Engineering, Tongmyong University, Busan 48520, Korea; msamin@tu.ac.kr; 2 Department of Electronic Engineering, Tongmyong University, Busan 48520, Korea

**Keywords:** deep learning, sentiment analysis, deep fusion, human activity analysis, disastrous situations analysis, social media

## Abstract

A rapidly increasing growth of social networks and the propensity of users to communicate their physical activities, thoughts, expressions, and viewpoints in text, visual, and audio material have opened up new possibilities and opportunities in sentiment and activity analysis. Although sentiment and activity analysis of text streams has been extensively studied in the literature, it is relatively recent yet challenging to evaluate sentiment and physical activities together from visuals such as photographs and videos. This paper emphasizes human sentiment in a socially crucial field, namely social media disaster/catastrophe analysis, with associated physical activity analysis. We suggest multi-tagging sentiment and associated activity analyzer fused with a a deep human count tracker, a pragmatic technique for multiple object tracking, and count in occluded circumstances with a reduced number of identity switches in disaster-related videos and images. A crowd-sourcing study has been conducted to analyze and annotate human activity and sentiments towards natural disasters and related images in social networks. The crowdsourcing study outcome into a large-scale benchmark dataset with three annotations sets each resolves distinct tasks. The presented analysis and dataset will anchor a baseline for future research in the domain. We believe that the proposed system will contribute to more viable communities by benefiting different stakeholders, such as news broadcasters, emergency relief organizations, and the public in general.

## 1. Introduction

Human activity analysis aims to analyze, recognize, and classify the physical actions performed by an individual (e.g., standing, walking, running, etc.). Sentiment analysis aims to extract and evaluate an individual’s views, thoughts, and facial expressions response about an entity (e.g., object, service, or activity). Sentiment analysis is extensively adopted by organizations to help them understand the views of customers regarding their commodities and services. The aim of human activity detection is commonly used to evaluate either medical diagnosis or abnormal activity based on data obtained from wearable devices or accelerometer readings. A recent example is reported by Bevilacqua et al. [1] where the raw data obtained from sensors were used to classify human activity. Researchers have been able to expand the reach of sentiment analysis to other fascinating applications through the recent growth and rise of social media [2]. This recent study reported the application of computational sentiment analysis to extract public sentiments from leading social media platforms about the Syrian refugee crisis. A second example is reported by Ema et al. [3] in which the neutrality of social media posts about the Austrian presidential election winner was evaluated and contrasted with the social media content of the rivals. 

The notion of human sentiment and activity analysis has been commonly used in NLP (Natural Language Processing), where a variety of techniques have been used to derive optimistic, pessimistic, and neutral perception emotions and activities from text streams or sensors raw data. Due to significant development in NLP, in different application fields, such as sports, education, hospitality, and other businesses, an in-depth study of text streams from diverse sources is possible [4]. There have lately been several proposals by researchers to extrapolate information from visual content about human activities and related sentiments as separate problems. An overwhelming amount of visual sentiment analysis research focused on facial close-up image data, where facial features are used as visual markers to infer emotions [5]. Similarly, human activities have been largely deduced from nonparametric representations which are referred to as Part Affinity Fields (PAFs), which learn associated body parts of individuals in images [6]. Efforts are being made to expand the visual approach to a more complicated visual context, for instance, background and context details and multiple objects. In this area of study, the recent developments in deep learning have also greatly enhanced the outcomes [7]. Nevertheless, it is not straightforward to extract sentiments/emotions as well as human activity information together from visual content and many aspects need to be addressed.

This work aims to discuss and analyze a challenging problem regarding human sentiments with associated physical activities in disaster-related images collected from social media platforms. People are increasingly using social media networks such as Instagram and Twitter to share situational alerts in the case of crises and emergencies, including news of missing or deceased people, damage to infrastructure and landmarks, calls for immediate concerns and assistance, and so forth. There are many forms of information on social networks, such as texts, images, and videos [8]. In this context, we emphasize visual sentiment and associated physical activity response analysis from disaster-related visual content that is a promising area of study that will help consumers and society in a diverse range of applications. To this end, we suggest a deep sentiment and associated activity analyzer fused with a deep human count tracker to track the number of people in disaster-related visual content. We discuss the preprocessing pipeline of human sentiments and associated physical activity response analysis starting from the collection of image data, annotation, and conclude with the development and training of deep models.

To the best of our knowledge, this is the first effort to develop a benchmark for deep learning-based sentiment and associated human activity analysis fused with a human count tracker in an occluded environment with a minimum number of identity switches in disaster-related videos and images. Images related to catastrophe situations are complicated and contain multiple objects in an occluded environment (e.g., in floods, broken walls, fire, etc.,) with significant contextual details. We believe that such a complicated use-case is vitally important as an opportunity to address the processing pipeline of visual sentiment and associated activity response analysis and provide a framework for future research in the field. Additionally, human sentiment and associated activity analysis in disaster situations can have numerous applications that can contribute towards more welfare to the society. It can aid broadcasting media outlets to cover emergencies where people are at risk from a different point of view. Likewise, such a framework would be used by emergency relief organizations to disseminate information on a larger scale, based on the visual content expressing the actual number of people with their sentiments and associated activity response that best illustrates the facts of a certain incident. Besides that, a large-scale dataset is compiled, annotated, and made freely accessible to promote potential future research in the domain. The dataset, annotation sets, and trained weight files are made public for future research in this challenging domain [9]. A larger group task has been performed with a significant number of participants for the annotation of the dataset.

The key contribution of this paper can be outlined as follows:

- We diversify sentiment analysis to visuals and combined with associated human activity to a more difficult and critical problem of disaster analysis, typically requiring several artifacts and other relevant details in the context of images.
- We suggest deep learning models fusion architecture for an automated sentiment with associated human activity analysis based on realistic social media disaster-related images.
- We fused a deep human count tracker with the YOLO model that enables tracking multi persons in the occluded environment with reduced identity switches and provides an exact count of people on risk in visual content in a disastrous situation.
- Presuming that the proposed deep framework exhibits differently to an image by retrieving diverse but harmonize image features, we evaluate various benchmark pre-trained deep models separately or in combination.
- We conducted a crowd survey to annotate a disaster-related image dataset containing annotations for human sentiments and corresponding activity responses that are collected from social networks, in which 1210 participants annotated 3995 images.

The paper is arranged as follows: Section 2 concludes the literature review in three different domains such as sentiment analysis, human activity, and human trackers based on machine learning specifically deep learning. Section 3 describes the proposed methodology of the fusion of deep models for human sentiment and associated activity analyzer with deep human count trackers in disaster-related visual contents. Section 4 provides the statistics of the crowdsourcing study. It further evaluates the experimental results that are obtained from the crowd-sourced study and its implications on the results of deep model fusion. We further compare and evaluate our results with state-of-the-art deep learning benchmarks. Section 5 concludes the proposed methodology and discusses future directions in this research domain.

## 2. Related Work

In this section, we briefly review the previous related studies and split them into three main directions, such as analyzing human sentiment, analyzing human activity, and finally tracking and counting human activities. To avoid repetition, we quickly review the state-of-the-art, taking into account the diversity of the underlined procedure, system, input source, and maximum performance, as follows:

The analysis of sentiment relates to the use of machine learning conventionally natural language processing, text analysis, computational linguistics, and biometrics to systematically define, isolate, evaluate, and study affective states and subjective data. Natural language processing has contributed largely to the accurate determination of sentiment in text or spoken voices, regarding consumers’ assessments of products, movies, etc. [10,11,12]. The purpose of the study of sentiment is to decide whether the consumer’s text conveys a neutral, optimistic, or negative opinion. Currently, three approaches address the problem related to sentiments [13]: (1) machine learning methods, (2) lexicon based methods, and (3) hybrid approach.

**Figure 1:**
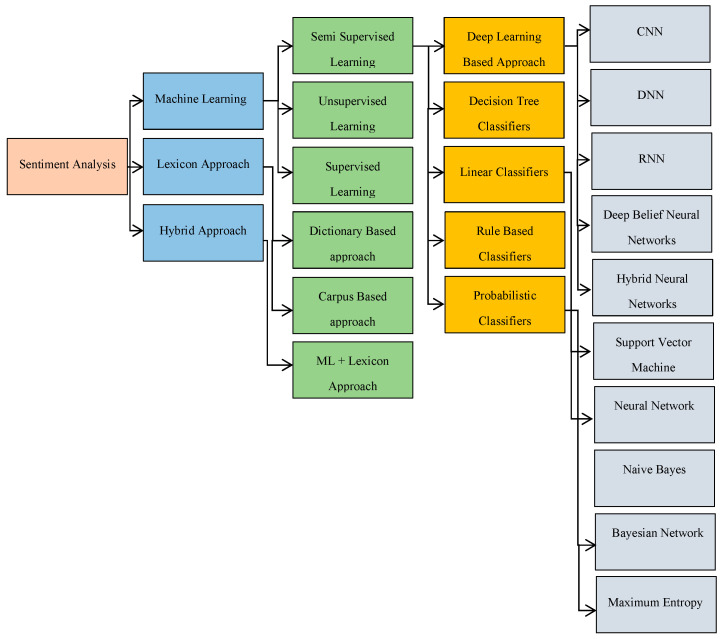
The taxonomy of sentiments analysis.

Machine learning methods can be split into two subcategories: (1) conventional methods and (2) deep learning-based [14]. Conventional approaches apply to traditional machine learning techniques such as Naive Bayes [15] and support vector machines (SVM) [16]. Deep learning techniques infer better performance than classical machine learning techniques. Such techniques contain deep neural networks (DNN), recurrent neural networks (RNN), and convolutional neural networks (CNN) for sentiment analysis. These methods address classification problems on text, speech, and visual content. Lexicon based methods were first applied to sentiment analysis. These methods rely on a statistical analysis of the content based on documents using techniques such as K-nearest neighbors (KNN) [10] and hidden Markov models (HMM) [11]. The hybrid approach combines both machine learning and lexicon applied to classify sentiments [12]. The sentiment lexicon exhibits a vital role in these strategies. Figure 1 describes the taxonomy of sentiment analysis. The literature regarding speculation of sentiments from image data is rather limited [17]. Moreover, being the latest and challenging task, there is a paucity of public datasets which makes it harder to construct a benchmark that can lay the foundation of a firm state of the art. Priya et al. [18] proposed a model to lower the effective gap that extracts objects with the high and low level of background features of an image. These extracted features lead to better recognition performance. A study represented in [19] that extracted features based on art theory and psychology for the classification of the emotional response of an image. The extracted features were grouped by content, texture, and color to be classified by using a Naive Bayes classifier. The study achieved promising results at a time; however, the extracted features struggled to recognize the complicated correlation between human sentiments and image contents. Ref. [20,21] provide a comprehensive survey on sentiments analysis that summarizes the common datasets, the main characteristics of the datasets, the deep learning model applied to them, their precision, and the contrast of different deep learning models.

Hence, the recent studies showed the criterion to extract adjective–noun pairs (ANPs) like sorrow face or happy girl, which may be utilized to deduce the emotional sentiment of an image. Damian et al. [22] constructed a dataset that contained 3000 ANPs, aiming to contribute to the research community. They also proposed a set of baseline models that are used frequently to benchmark techniques based on APNs [23]. Zaid et al. [24] presented a study containing a technique for the prediction of sentiment emoticons from images. They trained SimleyNet [24] on a largely collected dataset from social media which contained 4 million images and emoticon pairs. Several researchers have suggested the incorporation of visual and textual data for sentiment analysis. For example, in [25], authors proposed Attention-based Modality-Gated Networks (AMGN) that employed the images associated with text for the analysis of emotions.

The most common high-level cues used in human action detection in still images are the body, parts of the body (limbs, legs, etc.), the position of limbs, and legs including background details. Most of the human activity recognition published literature consists of supervised learning [26,27] and semi-supervised learning [28]. In the case of human activity recognition, the deep models require large training data; to tackle this problem, the transfer learning approach has been thoroughly studied [29]. Since many promising outcomes have been obtained, a widely agreed issue is that it is very costly to annotate all the human activities, as human annotators and deep learning experts offer numerous efforts, and the probability of error still remains high [30,31]. Nevertheless, providing reliable and relevant details is a salient task in human physical activity detection. Table 1 outlines the details of prominent research studies that are focused on supervised learning.

In the recent study [41] to inspire consumers in their everyday operations, this work introduces a music context conscious recommender framework. Its primary goal is to prescribe the most suitable music for the customer to maximize the performance of the physical activity at the recommended time.

In the event of a disaster, people could find it more helpful to capture images of the situation and publicly share them to warn others about possible threats, harm to human life, or facilities [42]. To this end, visual content will deliver precise information on the severity and extent of the damage, a better understanding of shelter needs, a more precise assessment of current emergency operations, and easier identification of missing and wounded. Early studies explore the significance of analyzing social media visual content in diverse catastrophe/disaster situations, such as flooding [43], fires, and earthquakes [44,45], motivated by this phenomenon.

The presented literature studies in this section reveal the trends which are followed for human sentiments as well as activities that are considered individual issues so far. Most of the features related to human activities are extracted from sensor data. However, we believe ‘A picture is worth a thousand words’. In disastrous conditions, visual content does not just contain descriptions of human emotions, but may also express ample clues correlated with human activity. These cues, which reflect the emotions and sentiments of photographers, can evoke similar feelings from the viewer and may help to interpret visual content beyond semantic meanings in various fields of use, such as education, entertainment, advertising, and media.

## 3. Proposed Methodology

**Figure 2:**
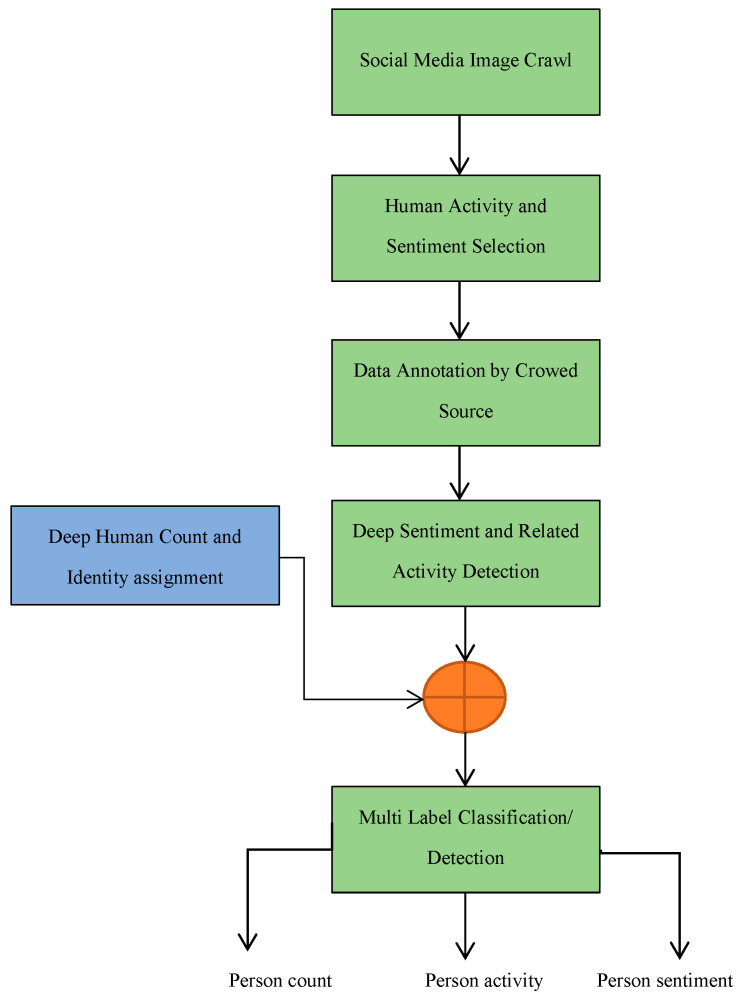
Block diagram of proposed system pipeline.

The proposed approach consists of seven steps in the pipeline. The block diagram in Figure 2 provides the architecture of the proposed methodology for visual sentiment and associated human activity analysis fused with a deep human count tracker.

The process begins with crawling social media networks for disaster-related images, preceded by the selection of accurate sentiment and associated human activities categorization by crowd resources. We manually analyzed collected images to remove irrelevant image data before proceeding towards the crowded source study phase. In the crowed sourcing phase, a subset of the image dataset is shared with human annotators for sentiment and related activity annotation. Next to the annotation phase, a Yolo based sentiment and human activity analyzer is used for the detection and classification of human activity and associated sentiment. This study aimed to uniquely detect and count human objects in an image as well as their sentiment and associated activity to analyze the exact situation in a disaster-related visual content. To aim this, another deep count CNN is used to count and uniquely identify humans in a particular image. The result of this deep count CNN is fused with Yolo outcomes to label human sentiment, associated activity, and the total number of humans in a visual scene using multi tags. In the subsection, we provide a detailed description of each component.

### 3.1. Data Crawling and Categorization of Human Activity with Associated Activity

The first phase starts with the collection of images from social network platforms such as Twitter, Google Images, and Flicker, etc. During crawling for images from mentioned social networks, the copyright aspect was under consideration and all those images were selected which were free to share. Furthermore, the images were crawled with specific tags such as floods, earthquakes, tornadoes, tsunamis, etc. These tags were made further detailed for locations such as ‘Nepal’s Earthquake’, ‘floods in Mexico’, ‘Tsunami in Japan’.

The selection for labeling the sentiments and associated activity is one of the crucial tasks in this research study. Most of the literature available concentrates on some specific sentiments such as ‘Negative’, ‘Positive’, ‘Neutral’ with no human-associated activity [46]. However, we intend to target sentiments that are more relevant to disaster-related content, taking into account the design and possible implementations of the suggested deep sentiment and associated human activity analysis processing pipeline. For example, labels such as ‘sorrow’, ‘excited’, and ‘anger’ are the sentiments that are more common in disaster-related situations. Moreover, according to a recent study about human psychology [47], we deduce two relevant sets of human sentiments that are more expected to be exhibited by people surrounded by a disaster. This study reported various types of human sentiments [29]. The first sets of sentiments include conventional human expressions such as ‘negative’, ‘Positive’, ‘Neutral’. The second list includes ‘Happy’, ’Sad’, ’Neutral’, ‘feared’.

The third set is the extended form of human expressions that included more detailed sentiments such ‘Anger’, ‘Disgust’, ‘Joy’, ‘Surprised’, ‘Excited’, ‘Sad’, ‘Pained’, ‘Crying’, ‘Fear’, ‘Anxious’, ‘Relieved’. The human activities associated with these sentiments are labeled as ‘Sitting’, ‘Standing’, ‘Running’, ‘Lying’, ‘Jogging’. Table 2 describes a detailed tagging of possible human sentiments and associated human activities in disastrous circumstances.

### 3.2. Crowdsourcing Study

For the proposed deep sentiment analyzer, the crowd-sourcing experiment aims to establish ground-truth by acquiring human views and thoughts about disasters and related visual information. The selected images were presented to participants conducted by Hireaowl [48] to annotate during the crowdsource study phase. In the process, a total of 3995 images were annotated by the participants. At least six individuals were selected to review the image to validate the consistency of the annotations. A total of 10,000 different answers from 2300 different participants were collected during the analysis. Individuals from multiple ages, gender groups, and 25 different countries were among the participants. The average response time by an individual is 200 s that helped us to filter out careless and inappropriate participants from the survey. Two trial tests were undertaken until the final analysis was done to fine-tune the test, correct mistakes, and improve consistency and readability.

**Figure 3:**
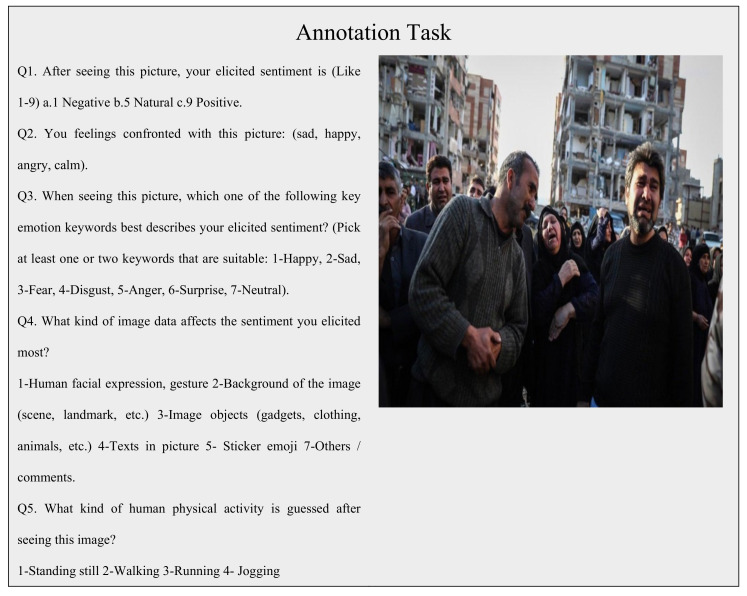
An overview of the web application used in the study of crowd-sourcing. The members who are asked to provide tags are presented with a disaster-related image.

Figure 3 shows a template of a questioner with a disaster-related image that was provided to participants to annotate human sentiment and associated activity. In the first question, the participants were asked to mark provided images in the range of 1 to 10, where 10 expresses ‘Positive’, 5 stands for ‘Neutral’, and 1 reflects ‘Negative’ sentiment. The objective of this question was to determine the general opinion of participants regarding the image. The second question is comparatively more specific such as ‘Sad’, ‘Happy’, ‘Angry’, and ‘calm’, which can extract the detailed sentiments that are conveyed by image to participants. In the third question, the participants were asked to tag the visual content from 1 to 7 and they can describe the sentiment that was conveyed to them by an image. Furthermore, the participants were asked to express their feelings about images that are provided to them and tag images manually about particular sentiment in case that sentiment tag is not present in the list of given tags. The fourth query attempts to highlight the features of the image that trigger human emotions and activity at the level of the scene or background context. In the fifth question, the participants were asked to express their perception about an associated human activity such as ‘Sitting’, ‘Standing’, ‘Running’, ‘Laying’, ‘Jogging’.

### 3.3. Deep Sentiment and Associated Activity Detector

**Figure 4:**
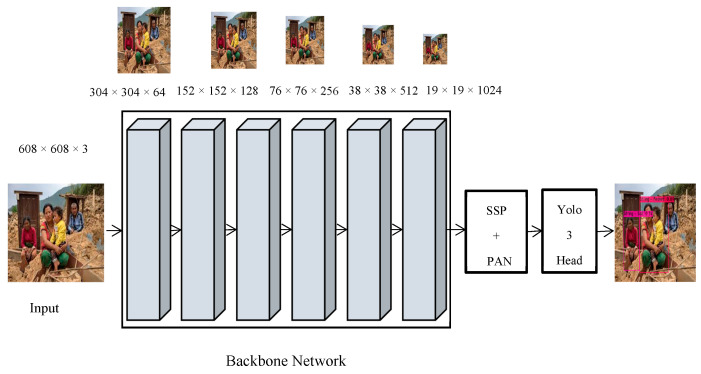
The network architecture of human sentiment and associated activity analyzer.

The architecture of deep sentiment and associated activity detector is shown in Figure 4. We utilized the CSPDarknet53 backbone which is a combination of Darknet 53 and CSPNet [49]. CSPNet was designed to solve the problems that require a lot of computation overhead at the network level. In network optimization, the problem of high inference computing is considered to be influenced by the duplication of gradient information, while CSPNet reduces computation by integrating gradient differences allover feature maps to ensure accuracy. It not only improves CNN’s learning efficiency, but it can also reduce the bottlenecks in computing and memory while retaining robust accuracy. Residual connections are used for the Darknet53 network module by leveraging the ResNet [50] principle of residues which address the network’s deep gradient issues. Two convolutional layers with a single shortcut link are used in each residue node, while the layers have several redundant residual modules.

The pooling layer and completely linked layers are not included in the architecture, whereas the network is under-sampled by specifying the convolution value as 2. After advancing over convolutional layers, the size of the image is reduced to half. Every convolutional layer includes convolution, Leaky ReLU, and batch normalization (BN), while, after each residual layer, zero-padding is applied. In addition, CSPDarnet53 Network Bone is connected to the SSP block, which produces a fixed size output independent of the input size by using different image dimensions as input to obtain pooling characteristics that have the length. As SPP is placed behind the last convolutional layer by just removing the existing pooling layer, it has no impact on the network architecture. As the SSP block is used with CSPDarknet53 network bone, the recognition range increases significantly, and it extracts the most important contextual features by affecting network operations even less to slow down. Table 3 describes the detailed insight of CSPDarknet 53 that is used as a component of human sentiment and associated activity analyzer in the article.

In addition to SSP, Path Aggregation Network (PAN) [51] is used as a parametric polymerization mechanism for various bone and detector levels instead of Feature Pyramid Network (FPN) [52] in the Yolo fourth version. It is a bottom-up path enhancement that seeks to promote the flow of data that can contribute to the optimization of the pyramid of features. PANET designed adaptive feature pools to connect the feature grid to all feature layers to allow useful information from each feature layer to transfer directly to the suggested sub-network beneath. Yolo3 is utilized as an anchor-based detection model network head [53]. In addition to these, the residual connections in the deeper Yolo3 mold network structure infer multi-scale detection that enhances mAP and greatly increases small object detections.

#### Loss Function

We have used the loss function Distance-IoU (DIoU) to reduce the normalized distance between the anchor box and the target box. It obtains a greater convergence rate and is more reliable and rapid when overlapping or even when using regression with the target box. It is concentrated on the union intersection (IoU) that takes the center distance of bounding boxes into account. Equation (1) describes the IoU where Bpred represents the prediction bounding box and Bgt represents the ground truth bounding box. Thus, Equation (2) represents the loss function in case the bounding boxes overlap. When the gradient doesn’t vary, the bounding boxes don’t overlap:

(1) $$IoU=\frac{Bpre\cap Bgt}{Bpre\cup Bgt}$$

(2) $$L_{IoU=1-}\frac{Bpre\cap Bgt}{Bpre\cup Bgt}$$

Therefore, GIoU improves the loss of *IoU* in the instance where the gradient does not alter without overlapping boundary boxes, which adds a penalty dependent on the *IoU* loss function. In Equation (3), an extra parameter C is added that reflects the minimal boundary box that can occupy both *Bpred* and *Bgt*:

(3) LGIoU=1−IoU+C−Bpre∩Bgtc

However, if, for example, the other box is overridden by either *Bpre* or *Bgt*, the penalty does not operate which is then called an *IoU* loss. DIoU is introduced to solve these limitations which are represented by Equation (4). The central point of the anchor box and the target box are represented by Bpre, Bgt, and *C* reflects the diagonal distance of the smallest rectangle that can fill the anchor and the target bounding boxes at the same time, while *p* is the Euclidean distance between two central points. Equation (5) represents the DIoU loss function:

(4) $$R_{DIoU}=\frac{p^{2}\left(Bpre,Bgt\right)}{C^{2}}$$

(5) $$L_{DIoU}=1-IoU+\frac{p^{2}\left(Bpre,Bgt\right)}{C^{2}}$$

DIoU loss function can apply non-maximum Suppression to eliminate the redundancy of the detection box. Not only the intervening area but also the difference between the ground truth detection box and the center point of the target box are taken into account, which would essentially avoid the above loss function flaws.

### 3.4. Deep Count and Identity Assignment

We adopted a conventional single hypothesis monitoring methodology of repetitive Kalman filtering and data association for human count and tracking. The framework for the count and track handling is mostly close to the original implementation in [54]:

(6) $$D={\left[x,\;y,\;s,\omega ,\dot{x},\;\dot{y},\dot{s},ώ\right]}^{t}$$

where *x* represents the target’s horizontal center and *y* depicts vertical pixel position, while *s* and ω represent the scale (area) and the aspect ratio of the target bounding box. It should be noted that the aspect ratio remains constant. The identified bounding box is used to optimize the target state where the obtained values are efficiently resolved while detection is paired with a target using a Kalman filter [55]. If detection doesn’t correspond to the target, its state is predicted without correction using the linear velocity model. By calculating its new location in the current frame when assigning detections to existing targets, the bounding box geometry of each target is determined. The assignment’s cost matrix is then calculated as the distance from the current target between each BBX observed and all predicted BBX as the intersection-over-union. Hungarian algorithms [56] are used to resolve the assignment optimally. In addition, to reject assignments where the target overlap detection is smaller than the minimal intersection-over-union, the least intersection-over-union threshold is used. We find that short term occlusion caused by moving target is indirectly addressed by the BBX IOU distance. Explicitly, only the occluder is detected when an occluding object obscures a target since similar-scale detections are properly preferred by the intersection-over-union range. This means that both the occluder object identification is corrected while the obscured target is untouched, meaning that no assignment is made.

As objects join and leave the image, unique identities need to be correctly created. The tracker or counter is initialized using the BBX shape, with a movement value assigned zero. Because the movement at this stage is not observed, the velocity variable covariance is initialized with significant values, representing this ambiguity. In addition, the newest counter then performs a probationary phase in which detections must be correlated with the target to acquire adequate data to stop counting false positives. The numbers of counts or trackers are aborted until not detected for $T_{lost}$ frames. This avoids the infinite increase in the number of trackers and localization errors caused without detector corrections by long-term predictions. In contrast, productivity is promoted by the premature exclusion of lost targets. If human objects reappear, with a new identity, tracking would effectively resume.

To achieve this, a convolutional neural network has been employed in a large-scale person re-identification dataset [57] containing more than 1 million images with 1000 human pedestrians, making it well-matched in a people counting and tracking context for deep count learning. Table 4 depicts the architecture of CNN that is employed for human tracking and counting. We are using a large residual network with six residual blocks accompanied by two convolutional layers. The global dimensionality feature map 128 is extracted in a dense layer.

A final batch and *l2* normalization, map features on the unit hypersphere to be in line with the cosine appearance parameter. The network has 2,800,864 parameters in total and on Nvidia GeForce (Santa Clara, California, CA, United States) GTX 1080Ti GPU, and one forward pass of 32 BBX requires roughly 31 ms. Provided that there is a stable GPU available, this network is also well suited for online counting and tracking.

The results of both networks are overlapped in a manner in which multi-label detection can be ensured. The detection from the Yolo based network generates two labels for sentiment and associated activity, and the results couple with the deep count tracker network that generates an extra detection label for the number of humans in visual content. The object and scene-level features are based on the responses of the participants in the fourth query, where they were asked to highlight the image features that trigger their emotions and feelings; we believe that this approach is helpful in the classification of sentiments and associated human activity. Besides this, we employed state-of-art benchmarks model such as Dense Net [58], Alex Net [59], Inception Net [60], VGGNet [61], and ResNet [50] to our collected dataset. These models are fine-tuned on our largely collected dataset for sentiment and associated human activity classification tasks.

In addition, we made some adjustments in the framework to fit the pre-trained models for the task at hand for the multi-label analysis. As a first step, with the corresponding changes in the models, a vector of the ground truth having all the possible labels has been generated. Particularly, the top layer of the model has been changed by replacing the soft-max function with a sigmoid function to facilitate multi-label classification. The sigmoid function is useful since it expresses the outcomes in probabilistic terms for each label, whereas the soft-max function retains the probability rule and smothers all the values of a matrix into a set of [0, 1]. Similar improvements (i.e., the replacement of Softmax with the sigmoid function) are introduced in the formulation of the cross-entropy for the pre-trained models to be better tuned. We divided the dataset into 70%, 10%, 20% train, validation, and test dataset ratio simultaneously.

## 4. Experiments and Evaluations

In this section, a detailed analysis of the crowded source study and the experimental results are presented.

### 4.1. Crowed Source Analysis and Dataset

**Figure 5:**
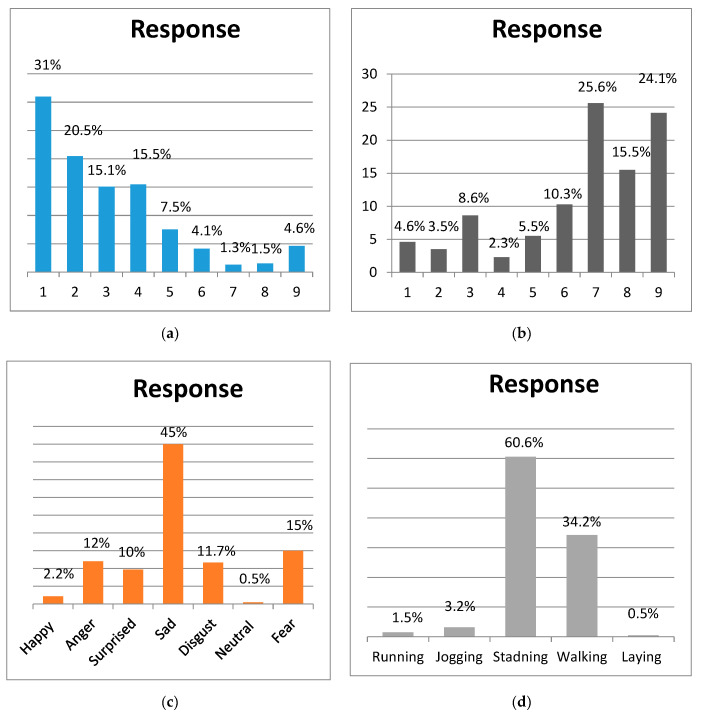
The statistics of crowdsource study: (**a**) statistics of the first question answers. Tags 1 to 9 tags 1 to 4 show negative emotions, while tag 5 is neutral positive feelings, and tags 6 to 9 indicate positive feelings; (**b**) answer figures for the second question. Tags 1 to 9, tags 1 to 4 indicate calm/relaxed emotion, tag 5 indicates natural state, while tags 6 to 9 reflect the state of excitement/stimulated; (**c**) statistics of question 4 answers; (**d**) statistics for associated human activity responses.

**Figure 6:**
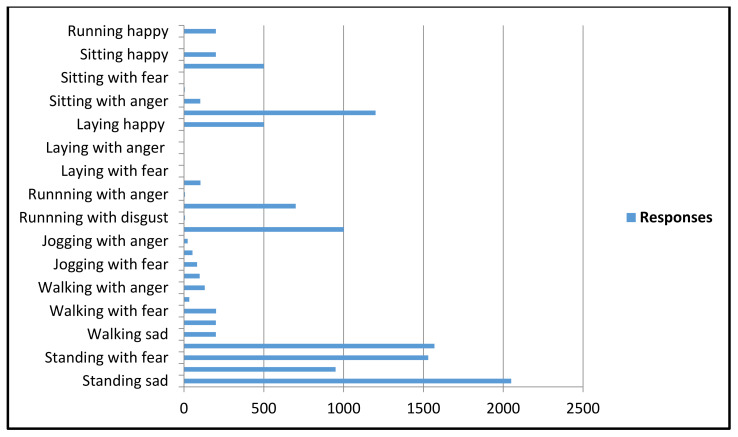
The statistics of joint sentiment with associated human activity response for Figure 3 by the crowdsource study.

Figure 5 depicts the detailed analysis of the crowded source study. The participants of this study were asked five questions that are described in Section 3. In the crowd-sourcing analysis, for example, the template image as shown in Figure 3 elicited pessimistic emotions by participants. Figure 5a (where tags 1 to 4 lead to negative feelings, 5 to neutral, and tags 6 to 9 represent positive feelings) indicates that most of the feelings are negative. Reaching the remaining answers, we observed that images were identified as positive during the relief and rescue process, but these responses are neglectable. In Figure 5b (where tags 1 to 4 indicate happy/excited, 5 neutral/calm, and 6 to 9 represent angry/sad), the responses ranged in a broad spectrum but almost of the answers resided in the sad indicator range. Figure 5c represents the response to the third question which contained a relatively larger range of options in the sentiment spectrum such as sad, surprised, anger, happy, fear, and neutral disgust. This sentiment spectrum helped participants as well as us to understand the more specific sentiment for a particular image. As expected, the responses were recorded as sad and feared by most of the participants. Figure 5d represents the related human activity as asked in question 5 from participants that can be visualized by a human observer by visual content. In the case of the sample image in Figure 3, a large number of participants responded with standing and a relatively fewer number of participants visualized the activity as walking as some of the image characters seemed to take the step of tending to move. The final question of the analysis, where we asked the participants to highlight the image sentiments with related human activity that affect their emotions, and tag selection for a given image is represented by Figure 6.

**Figure 7:**
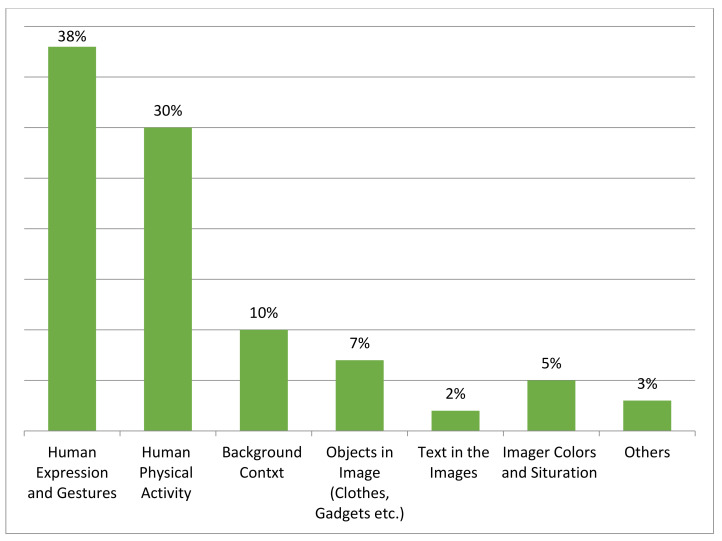
Overall statistics of crowdsource study about influential aspects in an image that evoked a human perception.

As expected, the responses by participants provided detailed perception about the visual content based on image context in a methodological manner. It is evident from Figure 7 that the background context information influences the evoking of common human perception about the actual scene to visualize the human sentiment with related physical activity (e.g., 41% human expression).

Human physical activity is very crucial (33%) besides human sentiments, and it is considered as correlated, as both influence each other. Other factors such as background context, text in image, image quality (contrast, saturation), and an object in images effectively evoked a human perception ranging from 11%, 2%, 5%, and 8%, respectively. Crowdsourcing study helped us in collecting an effective and meaningful dataset that contained human sentiment and associated activity images in disaster situations. Table 5 describes the detailed statistics of the dataset in terms of general sentiment distribution (e.g., positive, neutral, and negative). The statistics of a dataset that contain related human physical activity that is distributed in basic human physical reactions associated with sentiments (e.g., sitting, standing, walking, running, laying) are provided in Table 6. Table 7 provides detailed statistics of the breaking down of human sentiment into more expressive sentiment expressions. The images were then multi-labeled based on sentiments and associated human activity.

### 4.2. Experimental Results

The experimentations were conducted on Intel^®^ Xeon(R) (Santa Clara, CA, United States) that CPU contained 3.30 GHz octa-core processors with GPU GeForce (Santa Clara, CA, United States) GTX 1080Ti having 12 GB RAM. The Ubuntu 16.04 operating system was installed on the system.

**Figure 8:**
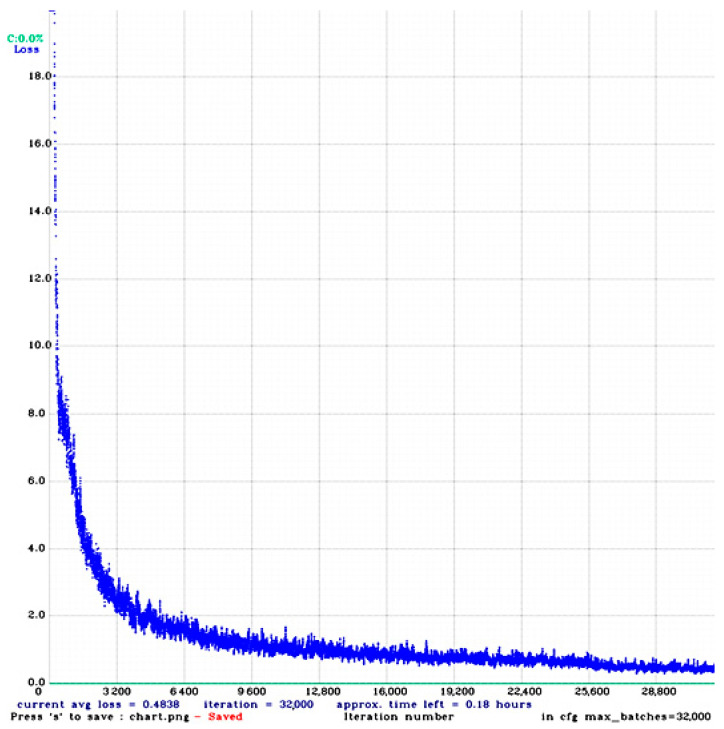
The summary of Yolo V4 based sentiment and associated activity training.

The model was fed with 608 × 608 × 3 (width, height, channels) image inputs. The batch size was set as 64 with 64 subdivisions due to limitations of GPU resources. The learning rate and momentum values were set as 0.001 and 0.949, respectively, while the value of decay was set as 0.005. We set the max-batch size with the formula (number of class × 2000), which is again a standard for using Yolo darknet version 4. For instance, if we have three classes, we must set the maximum batch as 6000. Next, we take 90% and 80% of the value of the max batch to generate optimal steps. In our case, the max batch value was set as 32,000, and the step value ranged between 25,600 and 28,800. Figure 8 depicts the summary of the training process where an average loss of 0.3 was achieved on our dataset.

**Figure 9:**
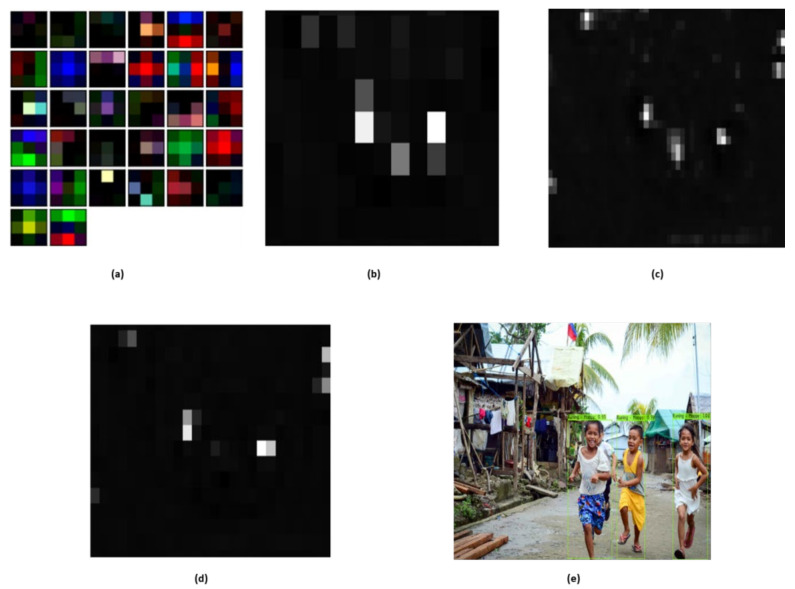
A general visualization of features detection of Yolo based sentiment and activity detector. (**a**) Obtained feature map from initial layer; (**b**) obtained feature map from layer 80; (**c**) obtained feature map from layer 85; (**d**) obtained feature map from layer 91; (**e**) Final Output.

Many literature papers aim to find the best accuracy that can be offered by a classifier and then present this value as the classifier’s efficiency. Seeking the best accuracy for a classifier though is typically not straightforward. In comparison, since this high precision can only be obtained with very particular data and classifier parameter values, the outcome is likely to be bad for another dataset, since the parameters have been calibrated for the specific data evaluated. Therefore, the classifier should perform well without being overly susceptible to changes in parameters, in addition to having high accuracy. That is, for a relatively wide range of values of its parameters, a good classifier can provide a stable classification [62]. It is challenging to clearly understand the mechanism of the deep neural network; however, we show several visual hints that DCNN could detect some discriminatory features. As can be seen from Figure 9, some filters have learned color characteristics, such as brown, red, green, etc., while some filters learn edge knowledge in multiple ways. To demonstrate the efficacy of the Yolo based sentiment and activity detection model, some of the feature maps obtained from various convolutional layers (80, 85, and 91) can be seen in Figure 9b–e. Some glimpses of feature detection of these layers are shown with different scales. The feature map in Figure 9a reveals that only the areas corresponding to the most important objects (kids) are activated. Although one object in two objects (kids) was obscured, the area of the middle kids was still weakly triggered. Incorporating the outcomes from various scales, the model correctly detects all the kids with face sentiments and associated activity.

A variety of experiments was conducted to test the efficiency of the proposed Yolo-based human sentiment and associated activity analyzer. The trained model evaluation indices are Recall, Precision, and F1 score.

Another evaluation metric that is used in this study is AP (average precision), typically finding an area under the precision–recall curve that can illustrate the model performance with confidence levels. AP is defined as in Equation (7) below:

(7) $$AP=\sum_{n}(r_{n+1}-r_{n}).P_{interp}\left(r_{n}+1\right)$$

where

(8) $$P_{interp}\left(r_{n}+1\right)=\;\underset{ȓ:ȓ\geq r_{n}+1}{max}.P\left(ȓ\right)$$

$P\left(ȓ\right)$ is a measured precision–recall curve.

Yolo v4 based sentiment and associated activity were evaluated with task 1 initially. Task 1 corresponds to only detecting the general sentiments of human objects such as positive, neutral, and negative in disastrous situations. Table 8 represents the results for task 1.

The second part of task 1 is based on human activity (sitting, standing, walking, running, laying) in disastrous situations. We have conducted separate experiments to detect human activity as single labeled problems like the task 1 sentiment analyzer. Table 9 shows the results for the Yolo based human activity analyzer.

We extended our experiments with multi-label and multi-task detection where we added extended sentiments (happy, excited, fear, anger, neutral, sad, disgust, surprise, relief) with associated human activity. Table 10 depicts the details of task 2. The experimental results that are shown in Table 10 depict every sentiment with associated activity results in a different score. For example, we can see that the given metric scores for activity lying are lower than other physical activities. The possible reasons can be (1) the human object faces are not visible when in the laying position. (2) The activity of human objects is static like most of the objects in the background context. (3) The number of specific sentiment images for training e.g., the number of images for disgust is lower so we found lower values of metrics for this sentiment.

Since one of the key reasons for the experiments is to provide a potential foundation in this domain, with many existing deep models, we compared and assessed the proposed multi-label framework with these benchmarked models. The obtained results from these benchmark deep learning models revealed that our chosen framework is a better option as compared to these models. These models only classify the sentiments and associated activities, but the Yolo based model performs one step further which detects human sentiments and associated activities in real time with a proper bounding box and multi-label captioning. This multi-label captioning is a more natural and reliable source to understand the human sentiment and associated activities in disastrous situations.

As shown in Table 8, we obtained the results in terms of accuracy, precision, recall, and F1 score for task 1 (positive, negative, and neutral) sentiments from these benchmark models pre-trained with datasets like Image Net. By comparing the contents of Table 8 and Table 11, it is evident that using the Yolo version 4 based sentiment analyzer yields better results for recognition and detection than other benchmark models. Similar experiments were conducted for human activity analysis in disaster situations using a deep learning benchmark model. It is evident from Table 12 that the Yolo based activity analyzer has performed better than these deep models—in particular, the effect of class labels smoothing, the influence of various data augmentation strategies, bilateral blurring, mix-up, cut mix and mosaic, and the influence of various activations, such as Leaky-ReLU, Swish, and Mish. The performance of the classifier is increased in our experiments by adding features such as cut mix and mosaic data augmentation, class mark smoothing, and triggering of Mish. As a result, the following features are included in our backbone for classifier training. Moreover, the dynamic scale of the mini-batch and the automatic rise in small resolution training, mini-batch size influenced improved detection for small-size items by using random training shapes.

We evaluate the performance of deep human count and tracking in disastrous related visual contents based on MOT16 (Moving Objects Tracking) [63] described as follows:

- MOTA (Multi-object tracking accuracy) is the primary metric that summarizes cumulative detection accuracy in terms of false positives, false negatives, and identity switches. MOTA can be described using Equation (9):
  (9) $$MOTA=1-\sum_{t}FN_{t}+FP_{t}+IDS_{t}$$
  where $FN_{t}$ represents missed targets and false positives (ghost paths) are $FP_{t}$, and the number of identity changes at time t is $IDS_{t}$. In case the intersection of union with the ground truth is inferior to a specified threshold, a goal is deemed missing. It is worth noting that the values for MOTA can be negative:
- MOTP (Multi-Object Tracking Precision) is a relative difference between all true positives and associated actual targets. This is determined as bounding box overlap, as:
  (10) $$MOTP=\frac{\sum_{t,i}d_{t,i}}{\sum_{t}c_{t}}$$
  where $c_{t}$ represents similarity in frame t and $d_{t,i}$, with its allocated ground truth object, the bounding box overlaps target *i*. Thus, MOTP presents the overall overlap for all correlative predictions and ground truth targets that scales from $d_{t,i}=$ 50 percent to 100 percent:
- MT (Mostly Tracked) is a portion of ground-truth records that have at least 80% of their life cycle under the same tag.
- ML (Mostly Lost) is a portion of targets that have a minimum 20% life span under the same tag.
- IDS (Identity Switches) are the number of changes or shifts in a ground-truth track’s recorded identity.

Based on these metrics, we evaluated the deep human count tracker framework. In Table 13, we have evaluated and compared the results with a deep human count analyzer with multiple techniques. The results show that the proposed deep human count is better than the mentioned techniques and is most suitable for real-time tracking.

**Figure 10:**
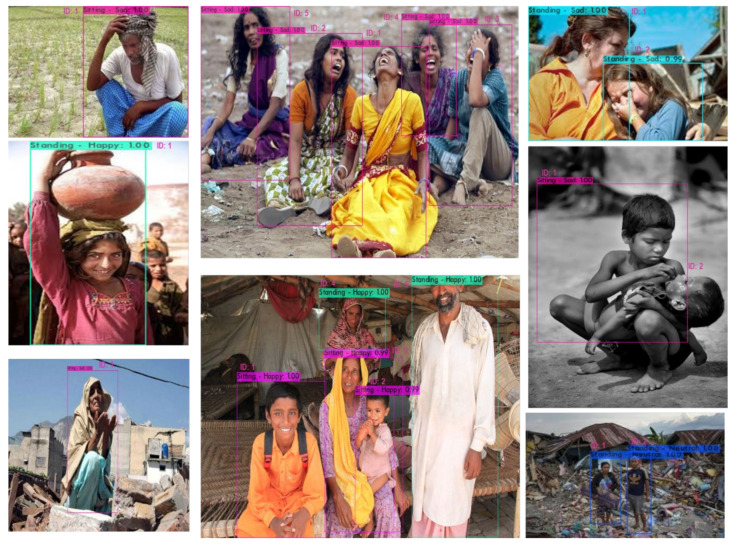
Multi-label sentiments and associated activity with unique human identity results from the proposed framework.

The results of deep sentiment and associated human activity analyzer (multi captioned) are then fused with deep human count (human ID in visual content). The result contains the human objects with unique identification numbers captioned on the detected bounding box with detected human sentiment and associated human activity captions as can be seen in Figure 10.

This preliminary study on the analysis of visual sentiments and associated activity analysis in disasters has uncovered several challenges, showing us all the various aspects of such a dynamic area of research. We have outlined the key points below:

- Human sentiment and associated human activity analysis in disastrous situations attempt to derive the perceptions about images from people; therefore, crowd-sourcing appears to be an effective option for obtaining a data set. Nevertheless, it is not straightforward to select labels/tags to perform an effective crowd-sourcing study.
- In applications such as disaster/catastrophe analysis, the three most widely used sentiment tags, namely positive, negative, and neutral combined with associated human activities, are not adequate to completely leverage the ability of visual sentiment and associated human activity analysis. The complexity surges as we broaden the spectrum of sentiment/emotion with associated human activities.
- The plurality of social media images associated with disasters reflects negative feelings (i.e., sorrow, terror, discomfort, rage, fear, etc.). Nevertheless, we realized that there are a variety of samples that can elicit optimistic feelings, such as excitement, joy, and relief.
- Disaster-related social media images display ample features to elicit emotional responses. In the visual sentiment study of disaster-related images, objects in images (gadgets, clothing, broken buildings, and landmarks), color/contrast, human faces, movements, and poses provide vital signs. This can be a key component in representing the sentiment and associated activities of people.
- As can also be observed from the observations of the crowdsourcing analysis, human sentiments and associated tags are linked or correlated, so a multi-label framework is likely to be the most optimistic direction of research.

## 5. Conclusions

We concentrated on the new concept of visual sentiment with associated human activity analysis in this article and demonstrated how images related to natural disasters invoke the sentiments and perceptions of people. To achieve this, we proposed a pipeline that starts from data collection, annotation by using a crowdsourcing study, followed by sentiment and an associated activity analyzer that is fused with a deep human count tracker, finally yields multi-tags that represent human sentiments and associated activity with unique identities for humans with fewer identity switches in occluded context and disaster-related visual content. We evaluated and annotated more than 3500 images with three distinct sets of tags in the crowd-sourcing analysis, resulting in four different datasets of different sentiment and associated human activity hierarchies. The three most commonly used sentiment tags, namely positive, negative, and neutral combined with related human activities, are not appropriate for applications such as disaster/catastrophe analysis to fully exploit the capacity to analyze visual sentiment and associated human behavior. When we extend the definition of sentiment/emotion with related human behaviors, the scope grows. Social network images relevant to disasters show enough functionality to evoke emotional reactions. Things in images provide vital signs in the visual emotion analysis of disaster-related images. This may be a central factor in reflecting people’s sentiment and related physical activities.

Based on our study, we conclude that the analysis of visual sentiments with associated human activity in general and the analysis of content relevant to natural disasters, in particular, is an exciting area of research that will support researchers and society in a diverse range of applications. The latest literature reveals a propensity to interpret visual sentiment in general images posted on social media by deploying deep learning techniques to derive visual cues dependent on an object and facial expression. We believe, nevertheless, that visual sentiment and associated human activity analysis can be applied to more complicated images, as also seen in this work, where many sorts of image features and details may be used jointly, such as object and scene-level features, human faces, movements, and poses. This approach contains greater potential as a baseline for numerous humanitarian and relief services and applications.

We believe there is still a lot to be explored in this direction, and this study offers a foundation for potential work in the domain. We tend to utilize most recent developments in adversarial training in affective computation and emotion processing, inspired by the continued progress and accomplishments associated with adversarial training in artificial intelligence. Further research initiatives aimed at exploiting the highlighted benefits of adversarial training have also been called to our attention. We would like to apply GAN (generative adversarial networks) techniques to generate deep fake disastrous images mixed real catastrophe visual content. We believe that training with such a dataset can affect the performance of such detection networks. If successfully applied, the new generation of robust affective computing and sentiment analysis techniques that are capable of broad in-the-wild implementation would encourage and facilitate these techniques. We would like to gather a multi-model dataset in the future where the text correlated with images utilizes visual features that contribute to the enhanced interpretation of visual sentiments and human activities.

## Figures and Tables

**Table 1:** Summary of classical machine learning techniques to recognize human activities.

Reference	Techniques	Approach	Input Source	Activity	Performance
Alex et al. [32]	Naive Bayes, SVM, MLP, RF	Human activity classification	Images	Walking, Sleeping, holding a phone	86%
Jaouedi et al. [33]	Gated Recurrent Unit, KF, GMM	Deep learning for human activity recognition	Video frames	Boxing, walking, running, waving of hands	96.3%
Antón et al. [34]	RF	Deduce high-level non-invasive ambient that helps to predict abnormal behaviors	Ambient sensors	Abnormal activities: Militancy, yelling, vocal violence, physical abuse Hostility	98.0%
Shahmohammadi et al. [35]	RF, Extra Trees, Naive Bayes, Logistic Regression, SV	Classification of human activities from smartwatches	Smartwatch sensors	Walk, run, sitting	93%
Abdulhamit et al. [36]	ANN, k-NN, SVM, Quadratic	Classification of human activities using smartphones	Smartphones sensors	Walking	84.3%
Štulien˙e et al. [37]	AlexNet, CaffeRef, k-NN, SVM, BoF	Activities classification using images	Images	Indoor activities: working on a computer, sleeping, walking	90.75%
Alsheikh et al. [38]	RBM	Deep learning-based activity recognition using triaxial accelerometers	Body sensors	Walking, running, standing	98%
Ronao et al. [39]	ConvNet, SVM	Classification of human activities using smartphones	Smartphone sensors	Walking upstairs, walking downstairs	95.75%
Bhattacharya et al. [40]	RBM	recognition of human activities using smartwatches based on deep learning	Smartwatch sensors (Ambient sensors)	Gesture-based features, walking, running, standing	72%

**Table 2:** The detailed list of human sentiment and associated activity used in the crowded souring phase.

Sets	Sentiment Tags	Activity Tags
Set 1	Negative, Positive, Neutral	Sitting, Standing, Running, Lying, Jogging
Set 2	Happy, Sad, Neutral, Feared	
Set 3	Anger, Disgust, Joy, Surprised, Excited, Sad, Pained, Crying, Feared, Anxious, Relieved	

**Table 3:** The architecture of human sentiment and associated activity analyzer backbone CSPDarknet53.

Layer	Filters	Output
DarknetConv2D		
BN	32	608 × 608
Mish		
ResBlock	64	304 × 304
2 × ResBlock	128	152 × 152
8 × ResBlock	256	76 × 76
8 × ResBlock	512	38 × 38
4 × ResBlock	1024	19 × 19

**Table 4:** The CNN architecture of the deep count tracker.

Layer	Patch Size	Stride	Output
Conv	3 × 3	1	32 × 128 × 64
Conv	3 × 3	1	32 × 128 × 64
Max Pool	3 × 3	2	32 × 64 × 32
Residual Block	3 × 3	1	32 × 64 × 32
Residual Block	3 × 3	1	32 × 64 × 32
Residual Block	3 × 3	2	64 × 32 × 16
Residual Block	3 × 3	1	64 × 32 × 16
Residual Block	3 × 3	2	128 × 16 × 8
Residual Block	3 × 3	1	128 × 16 × 8
Dense	-	-	128
Batch and *l2* normalization	-	-	18

**Table 5:** Detailed images statistics of used in all tasks.

Sentiment Tags	Number of Images
Positive	518
Neutral	480
Negative	2002

**Table 6:** Detailed dataset statistics used in human activity expressions.

Physical Activity Tags	Number of Images
Sitting	780
Standing	713
Walking	782
Running	708
Laying	17

**Table 7:** Detailed statistics of sentiment breakdown for task 2.

Sentiment Tags	Number of Images
Happy	413
Excited	105
Feared	608
Anger	92
Neutral	480
Sad	1123
Disgust	203
Surprised	180
Relief	200

**Table 8:** The sentiment analyzers’ average results for task 1.

Sentiment	Precision (%)	Recall (%)	F1 Score (%)	AP (%)
Negative	96.75	95.19	95.81	97.75
Neutral	94.21	94.02	94.98	97.62
Positive	96.52	95.84	95.26	97.76

**Table 9:** Human object activity analyzers’ average results for task 2.

Activity	Precision (%)	Recall (%)	F1 Score (%)	AP (%)
Sitting	95.20	95.04	95.91	97.02
Standing	96.21	96.01	96.46	97.62
Walking	96.35	95.93	96.02	97.36
Running	93.02	92.09	93.10	97.21
Laying	96.35	96.21	96.45	97.16

**Table 10:** Sentiments and associated human activity analyzers’ average results for task 2.

Sentiment	Metric	Activity				
		Sitting	Standing	Walking	Running	Laying
Happy/Joy	Precision (%)	98.21	97.02	95.71	90.53	89.01
	Recall (%)	97.51	96.90	95.10	91.23	89.00
	F1 Score (%)	97.76	96.95	95.13	90.15	88.15
Anger	Precision (%)	95.53	90.20	94.23	89.13	80.52
	Recall (%)	94.12	89.12	94.12	88.07	80.12
	F1 Score (%)	94.70	90.01	93.14	89.11	80.32
Fear	Precision (%)	97.73	96.23	93.54	91.23	92.13
	Recall (%)	97.19	96.05	92.78	91.11	91.89
	F1 Score (%)	96.46	96.12	92.17	90.52	91.78
Sad	Precision (%)	98.57	95.79	93.56	90.52	95.02
	Recall (%)	98.23	95.36	92.19	89.19	94.56
	F1 Score (%)	98.17	95.11	93.38	90.27	94.12
Neutral	Precision (%)	98.11	94.34	89.10	85.12	78.12
	Recall (%)	96.62	93.03	88.91	84.43	77.79
	F1 Score (%)	95.15	93.5	88.56	82.45	77.34
Disgust	Precision (%)	80.12	80.56	78.78	77.12	90.56
	Recall (%)	79.34	80.22	78.01	77.34	90.12
	F1 Score (%)	78.56	79.45	78.12	77.05	90.27
Surprise	Precision (%)	94.01	90.12	81.12	79.01	85.45
	Recall (%)	93.98	89.45	79.05	78.56	85.01
	F1 Score (%)	93.56	89.01	79.10	78.89	84.89
Relief/Relax	Precision (%)	96.12	96.23	95.29	92.78	90.12
	Recall (%)	96.00	95.13	94.84	92.19	90.14
	F1 Score (%)	95.78	96.04	95.11	92.25	89.34

**Table 11:** Assessment of the deep models’ sentiment analysis on task 1 (i.e., three classes of single-label classification, namely negative, neutral, and positive).

Model	Accuracy	Precision	Recall	F1 Score
ResNet-50	89.61	86.32	85.18	85.63
ResNet-101	90.01	87.79	86.84	86.43
Dense Net	85.77	79.39	78.53	78.20
VGGNet (Image Net)	92.12	88.64	87.63	87.89
VGGNet (Places)	92.88	89.92	88.43	89.07
Inception-v3	82.59	76.38	68.81	71.60
Efficient Net	91.31	87.00	86.94	86.70

**Table 12:** Assessment of the deep models’ human activity analysis on task 2 (i.e., five classes of single-label classification, namely sitting, standing, walking, running, and laying).

Model	Accuracy	Precision	Recall	F1 Score
ResNet-50	82.74	80.43	85.61	82.14
ResNet-101	85.55	79.26	85.08	81.16
Dense Net	81.53	78.21	89.30	82.27
VGGNet (Image Net)	82.56	80.25	84.51	81.80
VGGNet (Places)	89.88	88.92	88.43	89.07
Inception-v3	82.30	79.90	84.18	81.60
Efficient Net	82.25	80.83	82.70	81.39

**Table 13:** Comparison of human count and tracking algorithms.

Technique	MOTA	MOTP	MT	ML	ID	Runtime
[64]	68.2	79.4	41.0%	19.0%	933	0.7 Hz
[65]	71.0	80.2	46.9%	21.9%	434	0.5 Hz
[66]	62.4	78.3	31.5%	24.2%	1394	35 Hz
[67]	52.5	78.8	19.0%	34.9%	910	12 HZ
Proposed	61.4	79.1	32.8%	18.2%	781	40 Hz
